# Dynamic Adjustment Model of the Water Rights Trading Price Based on Water Resource Scarcity Value Analysis

**DOI:** 10.3390/ijerph18052281

**Published:** 2021-02-25

**Authors:** Xiao-yuan Wu, Feng-ping Wu, Fang Li, Xia Xu

**Affiliations:** 1 Business School, Hohai University, Nanjing 211100, China; sherrywu9211@163.com (X.-y.W.); xiaxu839546573@163.com (X.X.); 2 National Engineering Research Center of Water Resources Efficient Utilization and Engineering Safety, Nanjing 210098, China

**Keywords:** water rights trading, water resource scarcity value, water rights trading price, dynamic adjustment

## Abstract

The formation of the water rights trading price is an important part of the water rights trading system. In order to continuously and dynamically reflect the social–economic development changes and water resource scarcity, herein, we discuss the dynamic adjustment of the water rights trading price from the perspective of water resource scarcity value analysis. First, we constructed the water resource scarcity evaluation index system from the four dimensions of the water resource natural endowment, the water resource supply, the water resource demand, and the water environment, and then we constructed the water resource scarcity index calculation model of the transferor, the transferee, and the comprehensive water resource scarcity index calculation model of both parties of the trading. Second, by analyzing the 30 comparable water rights trading cases in China since 2016, we established the response function of the water rights trading price to the water resource scarcity index, and then we analyzed the impact mechanism of the water resource scarcity index on the water rights trading price. Third, based on the two factors of “the water resource scarcity value” and “the capital time value,” we constructed a dynamic price adjustment model of water rights trading for different adjustment factors, so as to adjust the water rights trading price scientifically. Finally, we took the water rights trading in Helan County (Helan) of Ningxia Hui Autonomous Region (Ningxia) as an example. The research shows that: (1) During the trading period of water rights in Helan, the water resource scarcity index rises, and the water rights trading price should be increased year-by-year. Additionally, there are certain differences in the water rights trading price changes with the adjustment of different elements. Among them, considering the adjustment of “the water resource scarcity value” element, the water rights trading price of Helan should be increased from 1.0940 to 2.8574 CNY/m³ during the water rights trading period; (2) there are differences in the water rights trading cost under different payment modes, among which the annual payment mode increased the most, i.e., from 2.7350 × 10^8^ to 7.4500 × 10^8^ CNY. This study suggests exerting a regulating effect of the water scarcity value on the water rights trading price, so as to promote the construction of a more equitable and long-term water rights trading market.

## 1. Introduction

### 1.1. Review of China’s Water Rights Trading Practice and Water Rights Trading System

In the past 20 years, there have been two active periods in China’s water rights trading. The first period was from 2000 to 2006, with typical cases including regional water right trading in Dongyang–Yiwu of Zhejiang Province, water taking right trading in Ningxia and Inner Mongolia, and water right trading for irrigation users in Zhangye city of Gansu Province. This stage marked the transition of China’s water rights trading from theory to practice. The second period was from 2012 to present, with typical cases including regional water rights trading in Luxi–Anyuan of Jiangxi Province, inter-city water rights trading of the Yellow River Mainstream League in Inner Mongolia Autonomous Region, and the transfer project of the water intake allocation index in the Dongjiang River Basin in Guangdong Province. This stage indicates that China’s water rights trading system is gradually improving. From the practice of water right trading in China, there are mainly two payment methods: One is one-time payment and the other is installment payment. In 2000, Yiwu city of Zhejiang Province (the transferee) bought out the permanent use right of 50 million m^3^ water resources per year from Dongyang city (the transferor) with a one-time payment of 200 million CNY. In 2015, Luxi County of Jiangxi Province (the transferor) signed the ShanKouYan Reservoir Water Rights Trading Agreement with Anyuan District People’s Government (the transferee) and the PingXiang Economic–Technological Development Zone Management Committee (the transferee). According to the agreement, the trading volume was set at 62.05 million m^3^, the trading period at 25 years, and the total trading cost at 2.55 million CNY per year, and they adopted the 10-year installment payment method, i.e., 255,000 CNY per year. Although the payment modes of these two cases were different, the calculation of the water right trading price does not fully reflect the change of the water resource scarcity value and the capital time value. We conclude that China’s current water rights trading price calculation has the following characteristics: (1) the current water rights trading price pricing methods implemented in China mainly include government-guided prices. Both parties negotiate pricing, engage in open bidding, and commission platform trading. (2) The formation of water rights trading prices mainly includes ecological compensation fees, economic benefit compensation fees, trading commissions, engineering construction costs, etc. In China’s different water rights trading practices, water rights trading prices change to varying degrees due to various factors in the actual trading process. (3) Water rights trading prices are often calculated based on the various factors values of the current level year, and remain basically unchanged during the water rights trading period. According to a survey, in the case of the water rights trading between Dongyang and Yiwu, in recent years, some people in Dongyang, i.e., the transferor, believed that water rights trading damaged their long-term interests, causing conflicts between the two sides and hidden dangers of social instability. The measurement of the water rights trading price does not objectively reflect the dynamic changes of water scarcity, and this is one of the main points of controversy.

Regarding China’s water rights trading, system construction is relatively behind trading practice. China’s first water rights trading case, i.e., Dongyang–Yiwu water rights trading, appeared in 2000. However, it was not until 2005 that China’s Ministry of Water Resources issued relevant system documents: “Water Rights System Construction Framework” and “Several Opinions on the Transfer of Water Rights by the Ministry of Water Resources,” the purpose of which was to regulate various behaviors in the use and circulation of water rights, to improve water resource utilization efficiency, and to solve China’s water shortage problem. After 2012, the Chinese government repeatedly devoted efforts to establishing and improving the water rights trading system, so as to optimize the water resource allocation by making full use of water rights trading. In 2014, the Ministry of Water Resources (MWR) issued the “Notice on Carrying out Water Rights Pilot Work,” which determined seven pilot provinces, providing experience and reference for promoting the construction of a water rights system in China. In 2016, the MWR issued the “Interim Measures for the Administration of Water Rights Trading,” which defined the forms of water rights trading, mainly including regional water rights trading, water withdrawal rights trading, and irrigation water users’ water rights trading, and also determined the corresponding specific responsibility ownership, operation procedures, supervision, and management methods of water rights trading. In 2017, the First Document of Chinese Government clearly proposed to “accelerate the construction of water rights and water market, promote the determination and trade of the water resources use right” [1]. In 2018, the National Development and Reform Commission, the Ministry of Finance, and another nine departments jointly issued the “Action Plan for Establishing A Market-Based and Diversified Compensation Mechanism for Ecological Protection,” which proposed to encourage and guide water rights trading and to improve the water rights trading platform. The construction of a water rights system indicates that China’s water rights management has entered a new developmental stage centered on the construction of a water resources property rights system. Such construction is mainly reflected in the standardization of the entire process of water rights trading, making specific provisions on matters related to tradable water rights, including scope and type, subject and duration, price formation mechanism, platform operation rules, etc. At the same time, a national-level water rights trading platform was built, which further standardized the water rights trading process.

Although China’s water rights trading practice and system construction have achieved remarkable results, judging from the actual operating conditions, there are still some shortcomings. First, China’s water rights trading is a trade of the right to use water resources, with the purpose of promoting the conservation, protection, and optimal allocation of water resources. However, China’s existing water rights trading periods are often long, and some water rights trading is even bought out by the transferee to obtain permanent use rights. The transferee can obtain the right to use the water for its trading volume within the water right trading period. After the trading period expires, the transferee can determine whether it needs to submit another trading application based on the actual situation. Second, there are still risks and hidden dangers in the price measurement and payment modes. In the process of calculating the water rights trading price, the trading price is generally negotiated by both parties of the trading based on the current water supply and demand situation. However, due to insufficient consideration of the scarcity of water resources in the future, the impact of such scarcity changes on the trading price is not included in the calculation of the trading price. Third, due to the long-term effective control of the price level by the Chinese government, the transferor often overlooks the capital time value that should be considered in the installment mode. In addition, most water rights trading in China relies on the promotion of local governments, on the transferor urgently needing to use the income from the water rights trading to improve people’s livelihood, or on making up for the investment gap of water-saving projects and other projects, which also leads to the transferor ignoring the impact of the capital time value on the water rights trading price to a certain extent. Lastly, China’s water rights management often formulates or perfects corresponding policies after a period of time when problems are exposed in water rights trading practice. Among them, the “Interim Measures for the Administration of Water Rights Trading” is still in the trial stage. There are no clear regulations on how to calculate the basic pricing for different water rights trading forms, on how to establish an adjustment mechanism for trading prices based on the water resource scarcity of both parties, or on how to adjust the trading costs according to the capital time price under different payment methods.

Determination of the water rights trading price is the most important aspect in forming fair and just water rights trading. Once the water right trading agreement comes into effect, both parties of the trading must fulfill it seriously. In other words, the parties must continue to perform the agreement until the trading period expires, even if the agreed trading price is lower due to insufficient consideration of future water resources scarcity changes when signing the agreement. In order to avoid this situation, it is necessary for both parties to accurately estimate the possible impact of future water resources scarcity value changes on the trading price when signing the trading agreement, and take this into account when negotiating the trading price. In China, it has become common knowledge that water is not a resource that can be exploited indefinitely. With the continuous development of the social economy, people’s understanding of the attributes and value of water resources is being constantly updated, and the traditional concept of “inexhaustible” has been gradually replaced by the concept of “water is a precious scarce resource.” With the increase in water resource scarcity value, if the trading price is only agreed by both parties of the trade according to the supply and demand situation of water resources at the time when the trading contract is signed, and remains unchanged for a long time, the invariable water rights trading price cannot reflect the real value of water resources, which may cause some economic losses to the transferor or even the third party. At the same time, the transferee may waste water resources due to paying lower costs, and even increase sewage discharge, which will result in the trading price losing the leverage for supply and demand regulation, even leading to disputes between the two parties. One of the main reasons for the undervaluation of water rights trading price is that the negotiators involved in the water rights trading negotiation lack understanding of the water resource scarcity value in the future. This study aims to scientifically analyze the impact of water resource scarcity value changes on water rights trading prices, and propose a more reasonable method for determining water rights trading prices. On the one hand, it helps the water rights trading negotiators to agree on a more reasonable water rights trading price, and on the other hand, it helps promote the establishment of a more stable water rights trading market.

### 1.2. Literature Review

On the issue of the water rights trading price, scholars at home and abroad have carried out a large number of studies and have proposed different pricing methods for water rights trading. Herein, we mainly focus on the cost pricing method, the shadow pricing method, the game pricing method, the real option method, and other methods. (1) The cost pricing method. This is a common method for studying water rights trading prices based on the cost of water rights [2,3,4]. Tian and Hu [5] established two models of regional water right negotiation pricing and water drawing right bidding on the basis of the full cost price. Erfani et al. [6] calculated the water rights price from the perspectives of the public water supply, agriculture, and energy based on a cost pricing model. Zhang et al. [7] calculated the upper limit of the water rights trading price in Ordos city. (2) The shadow pricing method. The shadow pricing method emphasizes that the water rights trading price should be determined based on the marginal benefit value. Wu and Li [8] obtained the equilibrium solution of water rights trading by constructing the shadow price model and by using the differential game model. Zhu et al. [9] obtained the theoretical water resource value of different water use departments in each river section of the Huai River Basin by ascertaining the shadow price of water resources. Liu et al. [10] obtained the optimal allocation of water resources by adopting the linear programming optimization method, and calculated the shadow price of water resources in the nine major river basins in China. (3) The game pricing method. Fu et al. [11] established a Bayesian model of water rights trading bidding and auction under the condition of incomplete information, and combined the theory of complex adaptive systems, obtaining a water resource allocation plan and trading price. Liu et al. [12] proposed that the government’s coordination ability and water demand coefficient in the quasi-market were the main factors affecting the trading price of water rights. Di et al. [13] and Degefu et al. [14] obtained the equilibrium price solution of water rights trading through the two-level dynamic game model. (4) The real option method. He and Gu [15] analyzed the trading price of water options based on uncertainty theory, which enabled the corresponding income to be obtained for industry and agriculture. Lee [16] analyzed the investment options of groundwater development projects under the uncertainty of water prices by using the monopoly real option model. (5) Other methods. Mosha et al. [17] analyzed the optimal trading volume and water rights trading price in a basin based on the regional optimization model of the ecological compensation mechanism. Xu and Liu [18] integrated the rainfall index into the final trading price of the water option and obtained the water rights trading price under the influence of rainfall fluctuation.

Scholars have carried out useful explorations of water rights trading pricing methods based on different methodological perspectives and have achieved fruitful research results. These theories and practical methods have laid a good foundation for the research of this study, but there are still some limitations. First, the existing literature on water rights trading pricing mainly focuses on the cost analysis method, game pricing method, option method, etc., to obtain the optimal or satisfactory static price. However, the static price ignores the dynamic change of the water resource scarcity value and the capital time value. Second, China’s water resources are owned by the country, but water resources are relatively scarce. The theoretical research on the water rights trading price is still in its infancy in China, so there is an urgent need to establish a theoretical system for calculating the trading price of water rights in line with China’s characteristics and water conditions.

### 1.3. Research Ideas of This Paper

Water rights trading is an important means of making full use of the scarcity value of water resources and of realizing the optimal reallocation of water resources, and scientific calculation of the water rights trading price is a basic element of water rights trading [19]. To provide a scientific and reasonable basic price that can be consulted and negotiated by both parties, based on an analysis of the water resource scarcity value, we established a dynamic adjustment model of the water rights trading price, so that the water rights trading prices can continuously and dynamically reflect the social–economic development and the changes of water resource scarcity. The basic ideas of this study are as follows:

(1) Construction of the water resource scarcity index (WRSI) function (model A), which aims to identify the water resource scarcity changes of the transferor, the transferee, and the two parties of the trade. First, we established the water resources scarcity evaluation index system based on elements of the water resource natural endowment, water resource supply, water resource demand, and water environment. Then, we constructed the WRSI function and calculated the WRSI of the transferor and the transferee by using the additive weighting method, and we established a comprehensive WRSI based on both sides of the trading. It should be noted that if both parties are in the same area, the transferor and the transferee have the same WRSI.
(3) Construction of the response function for water rights trading price to WRSI (model B), which aims to analyze the impact mechanism of WRSI on the water rights trading price. Due to the lack of “vertical” comparative data of the same water rights trading case over time, we adopted the “horizontal” comparative method. By collecting the basic information of 30 comparable water rights trading cases since 2016, we calculated the comprehensive WRSI and analyzed the changes in water rights trading prices. Then, based on these, we established the response function of water rights trading price to the comprehensive WRSI.
(3) Construction of the dynamic adjustment model for the water rights trading price (model C), which aims to propose a method to adjust the water rights trading price according to the changes of the water resource scarcity value and the capital time value. On the basis of case analysis, we predicted the change trend of the comprehensive WRSI in the water rights trading period, and we analyzed the impact of the comprehensive WRSI changes on the water rights trading price using model B. Considering the impact of the capital time value, we established the dynamic adjustment model of the water rights trading price, which includes the influences of the water resource scarcity value and the capital time value.

**Figure 1:**
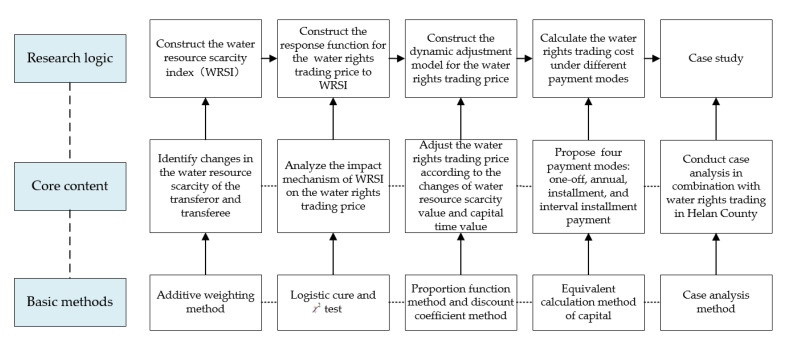
The basic idea of the study.

We also propose methods for calculating water rights trading costs for four payment modes: One-off, annual, installment, and interval installment payment modes. Then, we conducted case analysis in combination with water rights trading in Helan County (Helan), Ningxia Autonomous Region (Ningxia). The basic idea of the study is shown in Figure 1.

We conducted this work to promote the establishment of a fairer and long-term water rights trading market, to protect the legitimate rights and interests of both parties, and to reduce any disputes. The rest of this study is organized as follows: In Section 2, the study area is introduced; in Section 3, the methods are introduced; in Section 4, the results are analyzed; in Section 5, the study is concluded.

## 2. Case Study

**Figure 2:**
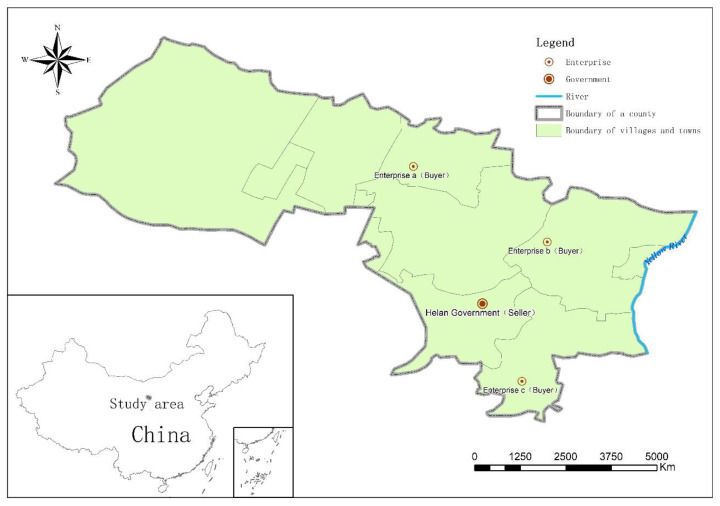
Geographical location of Helan County.

Helan of Ningxia is located in the central–northern part of Qingtongxia’s irrigated area. The geographical location of Helan is shown in Figure 2. The county is 8 km away from Yinchuan’s urban area, with a land area of 1595.5 km^2^. Its geographical coordinates are between 105°53′~106°36′ east longitude and 38°26′~38°48′ north latitude. The altitude of Helan Mountain in the west is more than 1400 m, with some areas as high as more than 3000 m. The piedmont floodplain at the eastern foot of Helan Mountain is 1122–1400 m above sea level, and the modern alluvial plain of the Yellow River in the East is 1102~1122 m above sea level.

In recent years, Helan has made great efforts to build a demonstration county in the comprehensive reform of the agricultural water price and modern ecological irrigation area, and has achieved remarkable results in water-saving renovation and construction. At present, Helan is transferring part of the use right of the Yellow River water through water rights trading, to further broaden the financing channels for the construction of modern ecological irrigation areas. We took Helan as a case study. The details of the water rights trading in Helan [20] are as follows:

(1) Transferor: Helan people’s government.
(2) Transferee: The enterprises that need to solve the industrial water consumption through water rights trading in Ningxia.
(3) Trading volume: The water rights trading volume is 10^7^ m^3^, which is the Yellow River water use rights saved by the implementation of water-saving renovation projects in Helan, and the trading volume is fixed at 10^7^ m^3^ per year.
(4) Trading period: Both parties of the water rights trade signed a 25-year treaty at one time, and the water rights trading in Helan will be implemented from 2020, so we set the trading period as 2021–2045.
(5) Trading price: The two parties agreed on a water right trading price of 1.094 CNY/m^3^, and the water right trading price is fixed within the trading period. According to “Analysis Report on Water Right Trading Index of Helan County,” the main basis for this price measurement include the infrastructure cost of the transferor under the current price level, the opportunity cost under the current water scarcity level, and the willingness and ability of the transferee to pay. Among them, the transferor’s infrastructure costs mainly include one-time investment in water-saving projects, accounting for 35.25%; the operation and maintenance costs of the project during the trading period, accounting for 17.76%; water-saving project renovation and renovation costs, accounting for 12.77%; the opportunity cost under the current water resources scarcity, which is mainly reflected in water rights compensation fees, accounting for 34.23%. When considering transferees’ willingness and ability to pay, the water rights trading price is basically at a low level in the same period in Ningxia. It can be seen from the price calculation that there are two obvious defects: One is that changes of water resource scarcity in the future are not taken into account; the other is that the variation in engineering and compensation costs with the rising price level or inflation in the future is not included.
(6) Payment mode: Enterprises can choose to pay the water rights trading cost year-by-year or as a one-time payment, according to their own operating conditions.

## 3. Methods

### 3.1. Evaluation Model of the Water Resource Scarcity Value

#### 3.1.1. Establishment of the Water Resource Scarcity Evaluation Index System

According to the absolute resource scarcity theory and relative resource scarcity theory, water resource scarcity also has the dual characteristics of absolute scarcity and relative scarcity. The natural endowment of water resources is the main source of its “absolute” scarcity. The per capita water resource occupancy in northern China is only 1/5 of the national average level and 1/20 of the world average level, representing a typical “resource-based water shortage” region. In southern China, due to the rapid development of the social economy in recent years, water resources are increasingly becoming “relatively” scarce. There are two main reasons for this. One is that the availability of water resources is reduced due to climate change, whereas the demand for water continues to increase, which leads to an imbalance between supply and demand, resulting in water resource scarcity, i.e., the demand for water resources exceeds the supply of water resources and causes water scarcity. This is called “the developing type of water shortage.” The other is that, with the continuous increase in the utilization of water resources, the discharge of sewage is also increasing day-by-day, and a large number of industrial sewage discharge fail to meet the discharge standards, which reduces the water quality of the river system and affects the quantity of available water resources, thus causing water resource scarcity. This type is called “water quality-induced water shortage.”

Based on the above analysis, we divided the factors affecting the water resource scarcity into four aspects: the water resource natural endowment, the water resource supply, the water resource demand, and the water environment. Among them, the water resource natural endowment and the water resource supply elements can reflect the influence of the external environment and climate change on the water resource scarcity value, while the water resource demand element can reflect the influence of water-saving technology on the water resource scarcity value through the change of the water resource utilization efficiency of agriculture and industry, and the water environment element can reflect the impact of the government’s water environment pollution control of the water resource scarcity value. In this study, a four-dimensional water resource scarcity evaluation index system was constructed, as shown in Table 1.

#### 3.1.2. Determination of the Water Resource Scarcity Index

(1) Data normalization

Considering that the dimensions of the different indicators may be different, the 0–1 linear transformation method was used to standardize the indicators, as follows:

For “+” type indicators:

(1) $$z_{tj}=\frac{x_{tj}-x_{\min}}{x_{j\max}-x_{j\min}}.$$

For “−” type indicators:

(2) $$z_{tj}=\frac{x_{\max}-x_{tj}}{x_{j\max}-x_{j\min}},\;$$

where $x_{tj}$ is the attribute value of index j in t year (including the current year and the planning year); $z_{tj}$ is the normalized attribute value; $x_{j\max}$ and $x_{j\min}$ are the possible maximum and minimum values of index j, respectively.

As for the determination of $x_{j\max}$ and $x_{j\min}$, different methods were adopted to determine the values according to the characteristics of different indicators. For indicators *C*_1_~*C*_5_, i.e., “natural factors,” the values were determined corresponding to the typical high-water areas and the typical arid areas. For indicators *C*_6_ and *C*_8_~*C*_16_, the values were determined corresponding to the typical technologically advanced areas and the relatively backward regions. For indicator *C*_7_, the values were determined corresponding to the actual maximum and minimum possible values.

(2) Determination of the indicator weight

The index weights were determined by the analytic hierarchy process method [21]. The water resource scarcity evaluation was taken as the target layer. The criterion layer was established from the four dimensions of the water resource natural endowment, the water resource supply, the water resource demand, and the water environment. The 16 indicators corresponding to the criterion layer constituted the indicator layer. Several relevant experts were invited to judge the importance of the criterion layer and the corresponding indicator layer based on the five-scale method. A pairwise judgment matrix was constructed based on the expert’s opinions. The elements in the matrix were approximately equal to the ratio of the weights of index h and index l. The root method was applied to obtain the relative weights of indicators under a single criterion as:

(3) $$\omega_{h}=\frac{\sqrt[{L}]{\prod_{l=1}^{L}a_{hl}}}{\sum_{h=1}^{L}\sqrt[{L}]{\prod_{l=1}^{L}a_{hl}}},\;$$

where *L* is the number of indicators under a certain criterion. According to Equation (3), after determining the relative weights of the indicators for each single criterion, the composite weight of each indicator relative to the target layer was calculated, set as $w_{j}$, j=1,2,⋯,16.

Following this, ten experts were invited, who were mainly from the China Water Rights Exchange, local water resources trading center, university professors, and the water administration department, to assign the importance degree of the indicators according to the five-scale method, and a pairwise judgment matrix was obtained. We obtained the weight $w_{j}$, j=1,2,⋯,16, of each indicator according to Equation (3), as shown in Table 2.

(3) Function of WRSI

The weighted method was used to construct the WRSI function. First, we calculated the WRSI of the transferor, as shown in Equation (4):

(4) $$R_{t}{}^{S\mathrm{eller}}=\sum_{j=1}^{16}z_{jt}^{S\mathrm{eller}}\cdot w_{j},$$

where $R_{t}{}^{S\mathrm{eller}}$ is the WRSI of the transferor in the t year and satisfies $R_{t}{}^{S\mathrm{eller}}\in \left[0,1\right]$.

Then, we calculated the WRSI of the transferee, as shown in Equation (5):

(5) $$R_{t}{}^{buyer}=\sum_{j=1}^{16}z_{jt}^{buyer}\cdot w_{j}$$

where $R_{t}{}^{buyer}$ is the WRSI of the transferee in the t year and satisfies $R_{t}{}^{buyer}\in \left[0,1\right]$.

We used the additive weighting method to construct the comprehensive WRSI function of both parties, as shown in Equation (6), which is recorded as model A:

(6) $$\begin{array}{cc} ModelA & \end{array}R_{t}=\theta R_{t}{}^{S\mathrm{eller}}+\left(1-\theta \right)R_{t}{}^{buyer}$$

where $R_{t}$ is the comprehensive WRSI of both parties in the t year and satisfies $R_{t}\in \left[0,1\right]$; θ is the proportion of the WRSI of the transferor, generally θ=0.5.

#### 3.1.3. Response Function of the Water Rights Trading Price to WRSI

In the practice of China’s water rights trading, the water rights trading price was determined by the two parties through scientific consultation. Considering that the existing water rights trading period is often long, the water rights trading price should be able to continuously and dynamically reflect the water scarcity changes and take into account the transferee’s affordability. In particular, we should fully consider the impact of the transferor’s water resource scarcity on the water rights trading price. When the transferor’s area is rich in water resources, the marginal utility of water rights is small, and the trading price can be appropriately reduced. In comparison, when the water resources in the area are scarce, the marginal utility of water rights is high, and only when the water rights transferee pays a higher trading price can water rights trading be promoted. At the same time, once the water rights trading price approaches the transferee’s affordability, the increase in the trading price will inevitably slow down.

To reveal the influence of water resource scarcity changes and the transferee’s affordability of the water rights trading price, and to provide evidence that water rights trading prices should be dynamically adjusted, we collected 30 cases of water rights trading in China’s water rights exchanges and local water resource trading centers since 2016. As a note, at present, due to the lack of longitudinal comparison data of the studied time dimension in the same case of water rights trading in China, we tried to analyze different cases horizontally to explore the change regularity of the water right trading price with the WRSI. In order to improve these cases’ comparability and to eliminate the interference with the water rights trading price effectively caused by the regional discrepancy and differences in water use, the 30 cases we screened had some similarities, i.e., they were all selected from the upper reaches of the Yellow River Basin; the water right trading took place in the internal or adjacent area; tradable water was mainly transferred from water-saving in agriculture to industrial enterprises.

The following steps were taken:

Step 1: Calculate the comprehensive WRSI, i.e., $R_{t}$. According to the evaluation index system designed in Table 1, we collected the index values of both parties of the trade in each case. For water rights trading in the internal area, only one side of basic information needed to be gathered because the transferor and the transferee were in the same region. The water resource scarcity evaluation indexes of each case were mainly derived from relevant national or regional policy documents, planning texts, the Water Resources Bulletin, etc. We used Equations (1) and (2) to standardize the evaluation index of water resource scarcity of the transferor and transferee. The weights determined in Table 2 were adopted to calculate the comprehensive WRSI $R_{t}$ in each trading case by using Equations (4)–(6). The WRSI $R_{t}$ of the different case are shown in Table 3.

Step 2: Analyze the water rights trading price changes in each case and calculate the water right trading price eigenvalue (WRTPE), i.e., $P_{t}^{E}$. The information on the water rights trading price mainly came from public information or related reports of the exchange and trading center. Due to the differences in the agreement years and regions of the 30 cases, the water rights trading prices fluctuate greatly in numerical terms. In order to increase the comparability of cases, and improve the test effect of the model in “Step 5,” we converted the water rights trading price of the 30 cases into the present value $P_{t}$ (unit: CNY/m^3^), and then used the interval number to convert this present value into the characteristic number in $\left[M^{0},M^{*}\right]$, which is called WRTPE, i.e., $P_{t}^{E}$. The specific algorithm is as follows:

(7) $$P_{t}^{E}=M^{0}+(M^{*}-M^{0})\frac{P_{t}-P^{\min}}{P^{\max}-P^{\min}},$$

where $P^{\max}$ is the maximum value of all the present water rights trading price values $P_{t}$, and $P^{\min}$ is the minimum value of all the present water rights trading price values $P_{t}$. We set $M^{0}=0,M^{*}=100$, and obtained the WRTPE of the different cases, as shown in Table 3.

**Figure 3:**
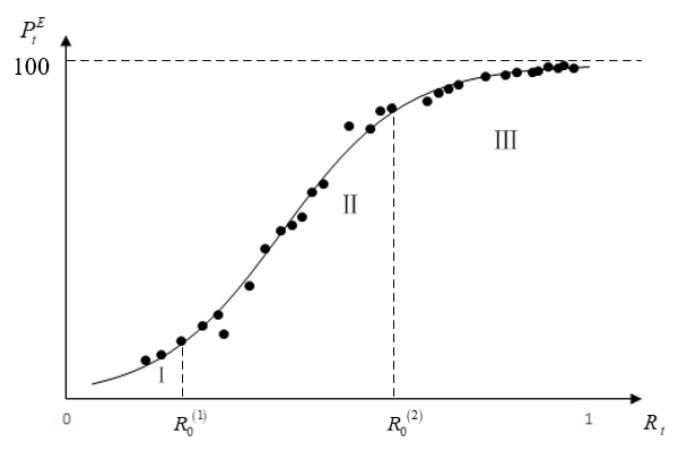
Sum scatter plot and fitting curve of the 30 cases of $R_{t}$ and $P_{t}^{E}$.

Step 3: Build the response function of the water right trading price to WRSI. It can be seen from the scatter plot of $R_{t}$ and $P_{t}^{E}$ in Figure 3 that the actual trading price is not in direct proportion to WRSI, yet these two have nonlinear characteristics of “slow-fast-slow” change. This indicates that in the process of water rights trading, water resource scarcity is a gradual process, resulting in gradual “growth” of the water resource value [22]. Therefore, a typical growth curve, i.e., a logistic curve, was selected to represent the changing trend of WRTPE, along with the WRSI, as shown in model B:

(8) $$\begin{array}{cc} ModelB & \end{array}P_{t}^{E}=1/\left(k+ab^{R_{t}}\right),$$

where a,b,k are the model parameters, satisfying a,b,k>0 and b≠1,0<b<1,k<1. We selected the appropriate model parameters to satisfy $P_{t}^{E}\in \left(0,100\right)$.

Step 4: Model solution. According to Table 3, the “three-section method” [23] (see Appendix A) was adopted to fit the model parameters, and the parameters were obtained as a=0.3677,b=0.0002,k=0.0109, i.e., the change of the model of WRTPE with WRSI was determined, as shown in Equation (9):

(9) $$P_{t}^{E}=1/\left(0.0109+0.3677*0.0002^{R_{t}}\right).$$

Step 5: Model test. We set hypothesis H_0_ as: The change of WRTPE with WRSI conforms to Equation (9). The goodness of fitness of the model was tested by the chi-square test method using the actual and predicted values [24], as shown in Equation (10):

(10) $$\chi^{2}=\sum_{i}^{h}\frac{{\left(A_{i}-E_{i}\right)}^{2}}{E_{i}},$$

where $A_{t}$ represents the actual value of WRTPE in the t,t=1,2,⋯,h year, i.e., $P_{t}^{E}$ in Table 3, and $E_{t}$ represents the predicted value of WRTPE in the t,t=1,2,⋯,h year, which was calculated using Equation (9). If $\chi^{2}\geq \chi_{0.05}^{2}$, then H_0_ could be rejected at the 0.05 level, while if $\chi^{2}<\chi_{0.05}^{2}$, H_0_ could fail to reject at the 0.05 level. The value of $\chi_{0.05}^{2}$ was determined according to the $\chi^{2}$ table.

According to Equation (10), it could be calculated that $\chi^{2}=5.1691$. According to a degree of freedom equal to *n* − 1 = 30 − 1 = 29, we obtained $\chi_{0.05}^{2}=42.56$. It is easy to know that $\chi^{2}<\chi_{0.05}^{2}$ and thus H_0_ could fail to reject, i.e., we were able to use Equation (9) to predict the WRTPE.

A sum scatter diagram of the 30 cases and a function image of Equation (9) are shown in Figure 3.

In Figure 3, the growth of the water resource scarcity value was divided into three stages, with a tangent slope of approximately 1 as the dividing point, for which $R_{0}{}^{\left(1\right)}$ was the critical value of stages I and II, and $R_{0}{}^{\left(1\right)}=0.22$, $R_{0}{}^{\left(2\right)}$ was the critical value of stages II and III, and $R_{0}{}^{\left(2\right)}=0.62$. In these three stages, stage I is called the “low-order insensitive” growth stage. In this stage, because the scarcity of water resources was not prominent enough, the scarcity value in water rights trading was not fully reflected. In this stage, the value of WRTPE changed from approximately 0 to 14.85. Judging from the 30 cases selected in our study, three cases were located in this stage. Stage II is called the “sensitive” growth stage; in this stage, the scarcity of water resources was highly valued by both parties involved in trading, especially the transferor, who evolved from cherishing water resources to having super high expectations of the water resource scarcity value. In this stage, the value of WRTPE changed from approximately 14.85 to 78.30. Judging from the 30 cases selected in our study, 14 cases were located in this stage. Stage III is called the “high-order insensitive” growth stage; in this stage, the scarcity of water resources became very prominent, especially the loss of “opportunity benefit,” and an increase of the “water-saving cost” of the transferor may have further aggravated the transferor’s reluctance to sell water resources. At the same time, in the water rights trading process, the transferee approached its maximum affordability, and the transferee may not have accepted the high trading price, so it was difficult for the scarcity value of water resources to maintain sustained and rapid growth in the process of water rights trading. In this stage, the value of WRTPE changed from approximately 78.30 to 100. Judging from the 30 cases selected in our study, 13 cases were located in this stage.

### 3.2. Dynamic Adjustment Model of the Water Right Trading Price

#### 3.2.1. Basic Assumptions and Variable Symbol Setting

(1) Basic assumptions. We established a dynamic adjustment model of the water rights trading price based on the analysis of the water resource scarcity value, to obtain a fairer and reasonable price and to ensure the interests of both parties.

The basic assumptions are as follows: ① The two parties involved in trading complete the water rights trading process under the coordination and supervision of the government; ② both parties involved in water rights trading have a clear understanding of the water resource natural endowment, the water resource supply, and the demand of the other party; ③ the scarcity of water resources is the basis of the value of water resources, and a change of water resource scarcity will lead to a change of the water right trading price; ④ both parties agree on the water resource scarcity value and the capital time value; ⑤ the transferor’s expectations of the water rights trading price should not only consider the “opportunity income” caused by the change of the water resource scarcity, but also the affordability of the transferee, i.e., fully consider the possibility of concluding the trading; ⑥ during the trading period, both parties involved in water rights trading can accept the continuous and dynamic changes of the price, and agree on an acceptable payment method according to factors such as the transferee’s payment capacity and the transferor’s capital needs.

In addition, assumption ① shows that the dynamic adjustment model of the water right trading price can be used as a reference for government departments; ② is the basis of using the scarcity value to estimate the water rights trading price in this study; assumption ③ is the basis of Equation (9); assumption ④ is the basis of Equations (11)–(13); assumption ⑤ is the basis of the logistic curve, i.e., the water rights trading price will increase with an increase in water resource scarcity, but the increase will be smaller in a certain position; assumption ⑥ is the basis of the two parties being able to choose different payment modes through negotiation.

(2) Variable symbol setting. By setting the trading period of water rights as n, the trading volume of water rights in the t year is $q_{t},t=1,2,\cdots ,n$. If the annual trading volume is fixed, i.e., $q_{1}=q_{2}=\cdots =q_{n}=q$, the WRTPE in the current year can be set as $P_{{}_{current}}^{E}$, the corresponding WRTPE in the i trading year as $P_{t}^{E}$, the trading price of water rights in the current year as $P_{current}$, the price of the t trading year as $P_{t}$, and the annual discount rate within the trading period as r.

#### 3.2.2. Price Adjustment Model under Different Elements

The dynamic price adjustment model of water rights trading was analyzed based on the two factors of the water resource scarcity value and the capital time value, as follows:

(1) Adjustment based on the element of the water resource scarcity value. By using the proportion function, we adjusted the trading price according to the WRTPE, and the adjustment coefficient was set as $P_{t}^{E}/P_{current}^{E}$. Therefore, the adjustment coefficient of the water rights trading price was obtained, set as $\sigma_{t1}$, as shown in Equation (11):

(11) $$\sigma_{t1}=\frac{P_{t}^{E}}{P_{current}^{E}}.$$

(2) Adjustment based on the element of the capital time value, i.e., the water rights trading price in the t year, was adjusted according to the factor of the capital time value, set as $\sigma_{t2}$, as shown in Equation (12):

(12) $$\sigma_{t2}={\left(1+r\right)}^{t}.$$

(3) Comprehensive adjustment of the two elements, i.e., based on the water resource scarcity value and the capital time value, the dynamic adjustment model of the water rights trading price was constructed, as shown in Equation (13):

(13) $$\begin{array}{cc} ModelC & \end{array}P_{t}=P_{current}\cdot \sigma_{t1}\cdot \sigma_{t2}=P_{current}\cdot \frac{P_{t}^{E}}{P_{current}^{E}}\cdot {\left(1+r\right)}^{t},t=1,2,\cdots ,n.$$

Therefore, according to Equations (11)–(13), the water rights trading price in different years under different adjustment elements were calculated. This is the basis of the determination of the water rights trading cost.

#### 3.2.3. Water Rights Trading Costs under Different Payment Modes

On the basis of an in-depth investigation and combined with the existing common payment modes in China, we analyzed four payment modes. The calculation of the water rights trading cost under the four payment modes is shown in Table 4 (see Appendix B).

Therefore, according to Table 4, the total water rights trading costs under different payment modes were obtained.

## 4. Results and Discussion

### 4.1. Data Sources

(1) Sample data. The data were from 2018. The average value of several years for the indicators were chosen to reflect the water resource natural endowment. Social and economic indicators were mainly taken from or referred to the relevant statistical yearbooks of Ningxia or Helan. The average total water resources, total surface water, precipitation, evaporation, industrial wastewater discharge, domestic wastewater discharge, water function area compliance rate, total water supply, total water consumption, effective utilization coefficient of farmland irrigation water, and water consumption of 10^4^ CNY were mainly obtained from the Water Resources Bulletin and the government’s reports. According to the statistics, from 2012 to 2018, the average water consumption of Helan was 433 million m^3^, of which 399.9 million m^3^ was from the Yellow River, accounting for 92.2%, and 33.8 million m^3^ was from groundwater, accounting for the other 7.8%. Furthermore, agricultural and ecological water consumption in Helan was 418 million m^3^, accounting for 94.9% of the total water consumption. Therefore, the runoff of the Yellow River trunk stream represents the main source of socio-economic water in Helan, mainly serving agricultural and ecological purposes.

(2) Prediction of relevant indicators. The total population, total GDP, industrial added value, irrigation area, and urbanization rate during the trading period were mainly taken from or referred to the relevant policy documents and planning texts of Ningxia or Helan. The water supply was determined in accordance with the red line of the total water intake control stipulated in the strictest water resource management system. The water demand mainly refers to the relevant planning texts of Ningxia or Helan, and combined with the trend of industrial structure evolution, the effective utilization coefficient of farmland irrigation water, water consumption of 10^4^ CNY, and water consumption rate were determined by referring to the actual changes of Helan from 2012 to 2018 and the development law of similar areas. In view of space, the actual data of the year 2018 and the forecast data for several years in Helan are as shown in Table 5.

Data sources mainly refer to “Ningxia Water Resources Allocation Guarantee Plan (2016–2020),” “Ningxia Agricultural Irrigation Water Quota (2014),” “Ningxia Current Status of Agriculture in 2018 Report on the Results of Measurement and Analysis of the Effective Utilization Coefficient of Irrigation Water,” “Outline of the 13th Five-Year Plan for National Economic and Social Development of Helan County,” “Thirteenth Five-Year Plan for Water Conservancy Development of Helan County (2016),” and “Thirteenth Five-Year Plan for Agricultural Industry Development in Helan County (2016).”

### 4.2. Water Resource Scarcity Value in Helan

On the basis of querying existing statistical data, comparing existing research, and considering relevant experts’ suggestions, the $x_{j\max}$ and $x_{j\min}$ of each evaluation index were determined. The data were normalized by referring to Equations (1) and (2). Then, because the transferor and transferee in Helan are in the same area, by using the weights in Table 2 and based on Equation (4), the WRSIs of Helan in 2018 and from 2021 to 2045 were obtained, as shown in Table 6, i.e., $R_{t}$.

By substituting $R_{t}$ into Equation (9), the WRTPEs, i.e., $P_{t}^{E}$, of Helan from 2021 to 2045 were calculated, as shown in Table 7.

**Figure 4:**
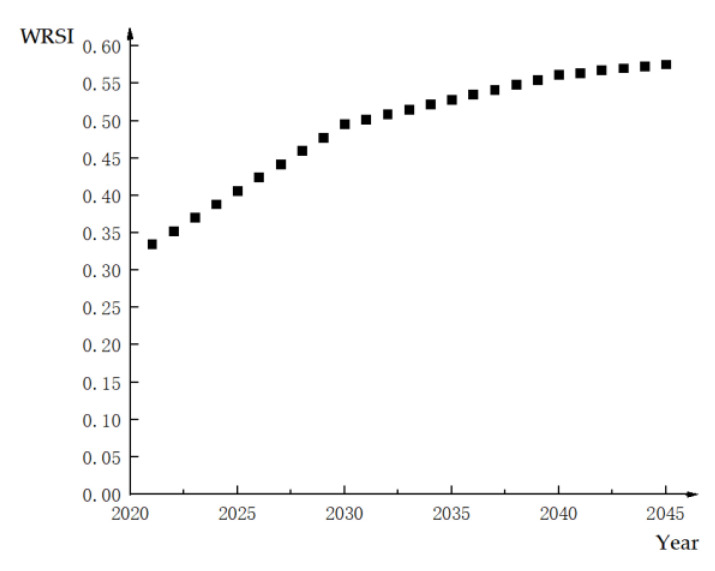
Change trend of WRSI in Helan.

**Figure 5:**
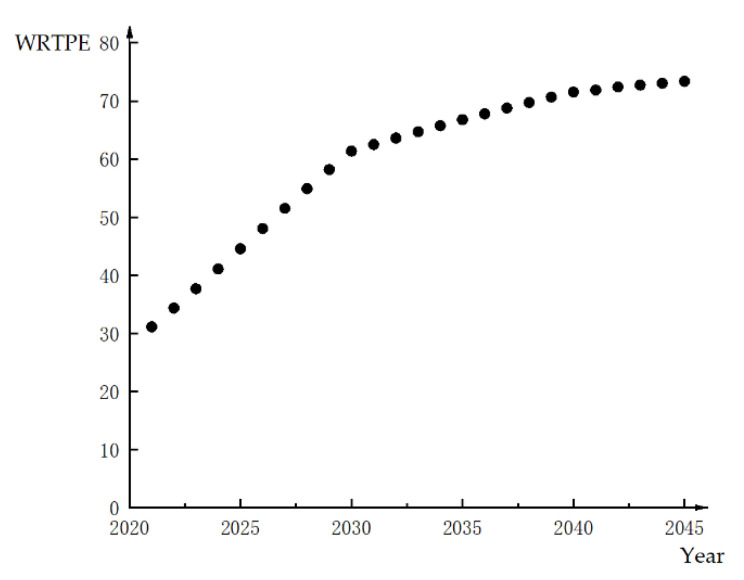
Change trend of WRTPE in Helan.

The variation trends of $R_{t}$ and $P_{t}^{E}$ in Helan from 2021 to 2045 are shown in Figure 4 and Figure 5, respectively.

It can be observed from Figure 4 and Figure 5 that the WRSI and WRTPE of Helan will increase year-by-year. This is consistent with the actual situation of Helan. Helan is located in an arid and water shortage area. With the development of the economy and society, the contradiction between supply and demand is prominent, and the pressure of the water environment is also great. Therefore, the water resources in Helan will continue to become increasingly “relatively” scarce. With the development of the economy and society, the scarcity of water resources has changed in the trading period. Therefore, it is necessary to recognize the value of water resources and to adjust the water rights trading price dynamically.

### 4.3. Adjustment of the Water Rights Trading Price in Helan

**Figure 6:**
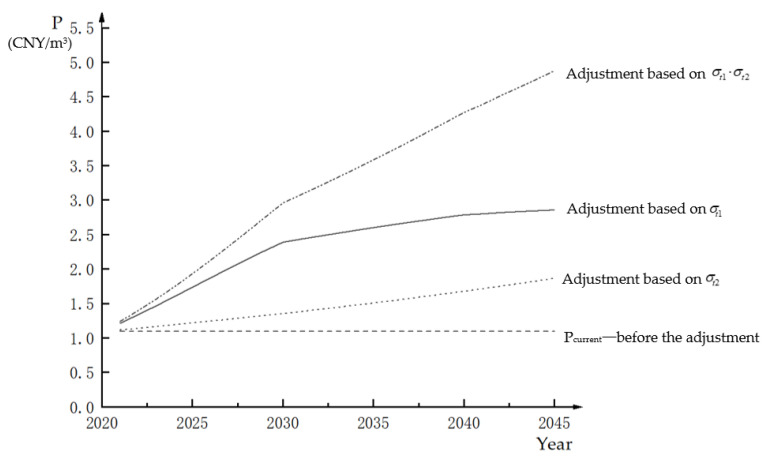
Dynamic change trend of the water right trading price in Helan from 2021 to 2045 under different adjustment elements.

The current trading price of Helan is $P_{current}=1.094\;\mathrm{CNY}/m^{3}$. The discount rate is r=2.16%, which was calculated according to the average consumer price index for several years. According to the WRTPE of Helan in different years, i.e., $P_{t}^{E}$ in Table 6, we obtained the adjustment coefficient, $\sigma_{t2}$ and the comprehensive adjustment coefficients $\sigma_{t1}\cdot \sigma_{t2}$ according to Equations (11)–(13). Then, the dynamic adjustment prices of water right trading in Helan from 2021 to 2045 were calculated, and the corresponding dynamic change curve was obtained, as shown in Figure 6.

It can be observed from Figure 6 that under the adjustment of different factors, the water rights trading price changes in Helan from 2021 to 2045 all show an upward trend, but the variation ranges are different. Specifically, the water rights trading price under the adjustment of two factors > the water rights trading price under the adjustment of the water resource scarcity value > the water rights trading price under the adjustment of the capital time value > the water rights trading price before the adjustment. Among them, under the adjustment of the water resource scarcity value, the water rights trading price changes from $P_{current}=1.094\;\mathrm{CNY}/m^{3}$ to $P_{2045}=2.857\;\mathrm{CNY}/m^{3}$. Under the adjustment of two factors, the price changes from $P_{current}=1.094\;\mathrm{CNY}/m^{3}$ to $P_{2045}=4.875\;\mathrm{CNY}/m^{3}$. Therefore, with the development of the economy and society and the change of the water scarcity value, the trading price of water rights will change greatly. If the trading price of water rights in Helan is fixed, with the understanding of the water resources value by both parties, disputes can easily arise in water rights trading.

### 4.4. Water Rights Trading Costs in Helan

The water rights trading volume of Helan is the same every year, i.e., $q_{t}=10^{7}\;m^{3}$. The water rights trading price is $P_{current}=1.094\;\mathrm{CNY}/m^{3}$, and the water right trading period is 25 years. Without considering the water resource scarcity value and the capital time value, the total water rights trading cost of Helan is 2.7350 × 10^8^ CNY. According to the four payment modes presented in Table 4, the water rights trading costs under different payment modes were calculated, as shown in Table 8.

It can be observed from Table 8 that under different payment modes, the elements of the water resource scarcity value all have an impact on the water rights trading cost in Helan. During the trading period, the WRSI of Helan presents an upward trend, and the water rights trading price should also increase year-by-year, which will lead to an increase in the water rights trading cost compared with that before the adjustment. In mode I, the transferee pays the water rights trading cost one time in 2021, and only needs to consider the influence of the water scarcity value. The water rights trading cost in Helan should increase from 2.7350 × 10^8^ to 5.8332 × 10^8^ CNY, and the water rights trading cost should increase by 3.0982 × 10^8^ CNY, indicating an increase of 113.28%.

Additionally, it can be observed that even though the water rights trading costs of Helan are different under different payment modes, they are all increased compared with those before adjustment. Compared with mode I, under modes II, III, and IV, in addition to adjustment of the factor of the water resource scarcity value, it is also necessary to consider the influence of the factor of the capital time value, which will increase the water rights trading cost in Helan. Under modes II, III, and IV, the water right trading cost of Helan is 7.4500 × 10^8^, 6.4100 × 10^8^, and 7.2014 × 10^8^ CNY, respectively, with an increase of 172.39%, 134.37%, and 163.30%, respectively. Among them, in mode II, due to the longest payment time span, the increase of water right trading cost is relatively large.

## 5. Conclusions and Recommendations

Based on the perspective of water resource scarcity value analysis, by considering the elements of the water resource scarcity value and the capital time value, a dynamic adjustment model of water rights trading prices under different element adjustments was established, and the water rights trading costs under different payment modes were discussed. The water rights trading in Helan was taken as an example in this study, and the results show that, during the water rights trading period in Helan, the price of water rights trading should increase year-by-year as the water scarcity value coefficient increases. A reasonable dynamic adjustment model that enables the water rights trading price to reflect the changes of social–economic development and the water scarcity continuously and dynamically could be established, to better protect the legitimate rights and interests of both parties of water rights trading. Additionally, there are differences in the water rights trading costs in Helan under different payment modes. For water rights trading with a long time period, the parties should negotiate and select a reasonable payment mode.

Based on the results obtained above, some recommendations are provided as follows:

(1) First, measurement of the water rights trading price should objectively reflect the dynamic changes of water scarcity, and both parties involved in water rights trading can negotiate to adopt reasonable payment modes according to their actual situations. Therefore, the water resource scarcity value should be coupled with the capital time value, and the water rights trading price should be calculated scientifically, to protect the legitimate rights and interests of both parties in water rights trading and to avoid the occurrence of disputes.
(2) Second, from the existing water rights trading cases in China, the trading period of water rights is often as long as 25 years, if not longer. If the trading period is too long, it will bring challenges to the prediction of relevant indicators, and the prediction error of indicators may directly lead to deviation of the measurement results of water rights trading price adjustment. To effectively avoid the impact of forecast deviation, we suggest that both parties involved in trading adopt mode IV, i.e., the interval installment payment mode. The specific interval period can be agreed upon by both parties according to the actual change of indicators. In this way, the WRSI and WRTPE can be calculated every five years according to the actual situation, and the trading price of the next five years can then be adjusted accordingly.
(3) Third, the legal system of China’s water rights trading market is not perfect at present. For example, evaluation and adjustment of the water rights trading price still lack clear supporting provisions. To promote the establishment of a standardized water rights trading market, we should improve the water rights trading price management system and establish a dynamic adjustment mechanism of the water rights trading price, to provide an institutional guarantee for dynamic adjustment.

It should be noted that in this study, when using Equation (6) to calculate the comprehensive WRSI of 30 cases, since the water rights trading of selected cases all happened within or between the same region, the WRSI of the transferor and the transferee was equal or close to each other, we could set θ=0.5. Due to the limitation of space, we did not make further sensitivity analysis on the value of θ. In the case analysis of Helan County, since the transferor and the transferee are Helan County, we directly use the data of the transferor to calculate the WRSI. When using the method in this study for example analysis, readers should conduct sensitivity analysis if the WRSI of the transferor and the transferee is significantly different. In other words, the comprehensive WRSI should be calculated by changing the value of θ, and then judge the influence of the change of θ on the trading price adjustment results, and obtain a relatively reasonable value of *θ*.

## Figures and Tables

**Table 1:** Index system of water resource scarcity evaluation.

Target Level	Criterion	Detailed Indicator	Measurement Method/Data Source	Label	Indicator Type
Evaluation of water resource scarcity	Water resource natural endowment	Per capita water resources (m^3^/person)	Total water resources/total population	*C*_1_	−
		Water production modulus (m^3^/km^2^)	Total water resources/total land area	*C*_2_	−
		Aridity index (−)	Evaporation/contemporaneous precipitation	*C*_3_	+
		Runoff depth (mm)	Total runoff/catchment area	*C*_4_	−
	Water resource supply	Water supply per capita (m^3^/person)	Available water supply/total population	*C*_5_	−
		Water supply rate (%)	Water supply/total water resources	*C*_6_	−
		Surface water supply ratio (%)	Total surface water/total water supply	*C*_7_	−
	Water resource demand	Per capita water consumption (m^3^/person)	Total water consumption/total population	*C*_8_	+
		Per capita domestic water consumption (L/person)	Total domestic water consumption/total population	*C*_9_	+
		Effective utilization coefficient of farmland irrigation water (−)	Net water consumption of crops/total water diversion from canal head	*C*_10_	−
		Water consumption of 10^4^ CNY (m^3^/10^4^ CNY)	Industrial water consumption/industrial added value	*C*_11_	+
		Urbanization rate (%)	Urban population/total population	*C*_12_	+
	Water environment	Water function area compliance rate (%)	From Water Resources Bulletin	*C*_13_	−
		Industrial pollution ratio (%)	Industrial wastewater discharge/total water supply	*C*_14_	+
		Life pollution ratio (%)	Domestic wastewater discharge/total water supply	*C*_15_	+
		Treatment rate of domestic sewage (%)	From Ningxia Statistical Yearbook	*C*_16_	−

Note: The index type “+” means that the greater the index value, the greater the degree of water resource scarcity, while “−” means the opposite.

**Table 2:** The weight of the water resource scarcity evaluation index.

**Indicator**	***C*_1_**	***C*_2_**	***C*_3_**	***C*_4_**	***C*_5_**	***C*_6_**	***C*_7_**	***C*_8_**
$w_{j}$	0.0821	0.0488	0.0282	0.0168	0.2499	0.0757	0.1376	0.1150
**Indicator**	***C*_9_**	***C*_10_**	***C*_11_**	***C*_12_**	***C*_13_**	***C*_14_**	***C*_15_**	***C*_16_**
$w_{j}$	0.0268	0.0169	0.0725	0.0441	0.0355	0.0251	0.0103	0.0145

**Table 3:** $R_{t}$ and $P_{t}^{E}$ of the 30 cases of water rights trading.

**Cases**	**1**	**2**	**3**	**4**	**5**	**6**	**7**	**8**	**9**	**10**
$R_{t}$	0.15	0.18	0.22	0.26	0.29	0.30	0.35	0.38	0.41	0.43
$P_{t}^{E}$	10.89	12.15	15.85	20.11	23.09	17.79	30.96	41.21	46.17	47.65
**Cases**	**11**	**12**	**13**	**14**	**15**	**16**	**17**	**18**	**19**	**20**
$R_{t}$	0.45	0.47	0.49	0.54	0.58	0.60	0.62	0.69	0.71	0.73
$P_{t}^{E}$	50.07	56.74	59.25	74.86	74.36	79.27	79.85	81.84	84.19	85.10
**Cases**	**21**	**22**	**23**	**24**	**25**	**26**	**27**	**28**	**29**	**30**
$R_{t}$	0.75	0.80	0.84	0.86	0.89	0.90	0.92	0.94	0.95	0.97
$P_{t}^{E}$	86.59	88.83	89.01	89.64	89.91	90.14	91.25	90.90	91.70	90.80

**Table 4:** Water rights trading costs under different payment modes.

Payment Mode	Interpretation of Mode	Water Rights Trading Costs
Mode I: One-off payment	Make a one-time payment at the beginning of the first year	The cost at the beginning of the first year is: $\sum_{t=1}^{n}\left[P_{current}\cdot \frac{P_{t}^{E}}{P_{current}^{E}}\cdot q_{t}\right]$
Mode II: Annual Payment	Make an equal payment at the beginning of each year	The cost at the beginning of each year during the trading period (*n*) is: $\frac{r{\left(1+r\right)}^{n-1}}{{\left(1+r\right)}^{n}-1}\cdot \sum_{t=1}^{n}\left[P_{current}\cdot \frac{P_{t}^{E}}{P_{current}^{E}}\cdot q_{t}\right]$
Mode III: Installment payment	Make an equal payment at the beginning of each year in the previous *m* years	The cost at the beginning of each year in the previous *m* years is: $\frac{r{\left(1+r\right)}^{m-1}}{{\left(1+r\right)}^{m}-1}\cdot \sum_{i=1}^{n}\left[P_{current}\cdot \frac{P_{t}^{E}}{P_{current}^{E}}\cdot q_{t}\right]$
Mode IV: Interval installment payment	Paying for the next five years at five-year intervals	The cost at the beginning of the *i*-th five years is: ${\left(1+r\right)}^{5\left(i-1\right)}\cdot \sum_{\theta =1}^{5}\left[P_{current}\cdot \frac{P_{\left(5i+\theta -5\right)}^{E}}{P_{current}^{E}}\cdot \frac{1}{{\left(1+r\right)}^{\theta -1}}\cdot q_{\left(5i+\theta -5\right)}\right]$

**Table 5:** Evaluation index value of the water resource scarcity in Helan.

Index	2018	2025	2030	2035	2040	2045
*C*_1_	1544.76	1544.76	1544.76	1544.76	1544.76	1544.76
*C*_2_	34.78	34.78	34.78	34.78	34.78	34.78
*C*_3_	8.90	8.90	8.90	8.90	8.90	8.90
*C*_4_	411,000	411,000	411,000	411,000	411,000	411,000
*C*_5_	1792.13	1729.40	1666.67	1641.67	1616.67	1605.67
*C*_6_	0.76	0.77	0.77	0.78	0.79	0.80
*C*_7_	0.86	0.86	0.86	0.86	0.86	0.86
*C*_8_	1684.37	1719.38	1754.39	1793.86	1833.33	1872.80
*C*_9_	181.00	186.50	192.00	197.68	203.35	209.03
*C*_10_	0.52	0.55	0.58	0.59	0.60	0.61
*C*_11_	13.00	21.50	30.00	32.50	35.00	37.50
*C*_12_	56.98	60.99	65.00	67.50	70.00	72.50
*C*_13_	0.79	0.82	0.85	0.87	0.89	0.91
*C*_14_	0.71	0.71	0.70	0.60	0.50	0.40
*C*_15_	2.72	2.61	2.50	2.45	2.40	2.35
*C*_16_	0.86	0.88	0.91	0.93	0.95	0.98

**Table 6:** The water resource scarcity index (WRSI) of Helan in different years ($R_{t}$ ).

**Year**	**2021**	**2022**	**2023**	**2024**	**2025**	**2026**	**2027**	**2028**	**2029**
$R_{t}$	0.3349	0.3528	0.3707	0.3885	0.4064	0.4242	0.4421	0.4599	0.4778
**Year**	**2030**	**2031**	**2032**	**2033**	**2034**	**2035**	**2036**	**2037**	**2038**
$R_{t}$	0.4957	0.5022	0.5088	0.5154	0.5220	0.5286	0.5352	0.5418	0.5484
**Year**	**2039**	**2040**	**2041**	**2042**	**2043**	**2044**	**2045**		
$R_{t}$	0.5550	0.5616	0.5641	0.5680	0.5706	0.5732	0.5757		

**Table 7:** The water rights trading price eigenvalue (WRTPE) of Helan in different years ($P_{t}^{E}$ ).

**Year**	**2021**	**2022**	**2023**	**2024**	**2025**	**2026**	**2027**	**2028**	**2029**
$P_{t}^{E}$	31.1424	34.3430	37.6680	41.0846	44.5558	48.0421	51.5034	54.9008	58.1982
**Year**	**2030**	**2031**	**2032**	**2033**	**2034**	**2035**	**2036**	**2037**	**2038**
$P_{t}^{E}$	61.3638	62.4934	63.6003	64.6834	65.7419	66.7749	67.7819	68.7622	69.7155
**Year**	**2039**	**2040**	**2041**	**2042**	**2043**	**2044**	**2045**		
$P_{t}^{E}$	70.6413	71.5395	71.8822	72.3955	72.7275	73.0553	73.3788		

**Table 8:** Water rights trading costs under different payment modes.

Payment Mode	Water Right Trading Costs
Mode I: One-off payment	The one-time payment at the beginning of 2021 is approximately 5.8332 × 10^8^ CNY
Mode II: Annual Payment	The cost at the beginning of each year during the 25-year trading period is approximately 0.2980 × 10^8^ CNY The total water rights trading cost is approximately 7.4500 × 10^8^ CNY
Mode III: Installment payment	The cost at the beginning of each year for the first 10 years is approximately 0.6410 × 10^8^ CNY The total water rights trading cost is approximately 6.4100 × 10^8^ CNY
Mode IV: Interval installment payment	The cost at the beginning of year 2021 is approximately 0.7021 × 10^8^ CNY The cost at the beginning of year 2026 is approximately 1.1352 × 10^8^ CNY The cost at the beginning of year 2031 is approximately 1.4933 × 10^8^ CNY The cost at the beginning of year 2036 is approximately 1.7911 × 10^8^ CNY The cost at the beginning of year 2041 is approximately 2.0797 × 10^8^ CNY The total water rights trading cost is approximately 7.2014 × 10^8^ CNY

## Data Availability

The data presented in this study are available on request from the corresponding author. The data are not publicly available due to the reports are for internal reference.

## Abbreviations

WRSI	Water resource scarcity index
WRTPE	Water rights trading prices eigenvalue
MWR	Ministry of Water Resources

## Appendix A

The idea of the “three-section method” is as follows:

First, according to Equation (6), to simplify the solution, we can set the function as $y=k+ab^{t}$. We divided the function into three parts, as shown in Equations (A1)–(A3):

(A1) $$\{\begin{array}{c} y_{1}=k+ab^{1}\\ y_{2}=k+ab^{2}\\ \vdots \\ y_{n}=k+ab^{n} \end{array},$$

(A2) $$\{\begin{array}{c} y_{n+1}=k+ab^{n+1}\\ y_{n+2}=k+ab^{n+2}\\ \vdots \\ y_{2n}=k+ab^{2n} \end{array},$$

(A3) $$\{\begin{array}{c} y_{2n+1}=k+ab^{2n+1}\\ y_{2n+2}=k+ab^{2n+2}\\ \vdots \\ y_{3n}=k+ab^{3n} \end{array}$$

Then, by adding the equations in Equations (A1)–(A3), respectively, we can obtain:

(A4) $$D=\sum_{i=1}^{n}y_{i}=nk+ab\frac{1-b^{n}}{1-b},$$

(A5) $$E=\sum_{i=n+1}^{2n}y_{i}=nk+ab^{n}\frac{1-b^{n}}{1-b},$$

(A6) $$F=\sum_{i=1}^{n}y_{i}=nk+ab^{2n}\frac{1-b^{n}}{1-b}.$$

By calculating (F−E)/(E−D), we can obtain the value of b, as shown in Equation (A7):

(A7) $$b=\sqrt[{n}]{\frac{F-E}{E-D}}.$$

By calculating (E−D) and substituting Equation (A7), we can obtain the value of a, as shown in Equation (A8):

(A8) $$a=\frac{{\left(E-D\right)}^{2}\cdot \left(1-\sqrt[{n}]{\frac{F-E}{E-D}}\right)}{\left(\frac{F-E}{E-D}-\sqrt[{n}]{\frac{F-E}{E-D}}\right)\cdot \left(2E-D-F\right)}.$$

By substituting Equations (A7) and (A8) into Equation (A4), we can obtain the value of k.

Then, we can obtain the function of $y=k+ab^{t}$.

## Appendix B

The calculation process of the water rights trading costs under different payment modes was as follows:

(1) Mode I: One-off payment

In this mode, according to Equation (10), we obtained the annual water rights trading price as follows:

(A9) $$P_{t}=P_{current}\cdot \frac{P_{t}^{E}}{P_{current}^{E}}.$$

Then, we obtained the water rights trading cost as shown in Equation (A10):

(A10) $$C=\sum_{t=1}^{n}P_{t}q_{t}=\sum_{t=1}^{n}\left[P_{current}\cdot \frac{P_{t}^{E}}{P_{current}^{E}}\cdot q_{t}\right]$$

(2) Mode II: Annual Payment

In this mode, by setting the cost at the beginning of each year during the trading period (n) as c, we obtained Equation (A11):

(A11) $$\sum_{t=1}^{n}\left[P_{current}\cdot \frac{P_{t}^{E}}{P_{current}^{E}}\cdot q_{t}\right]=c+\frac{c}{1+r}+\frac{c}{{\left(1+r\right)}^{2}}+\cdots +\frac{c}{{\left(1+r\right)}^{n-1}}$$

By solving Equation (A11), we obtained the cost at the beginning of each year during the trading period (*n*), as shown in Equation (A12):

(A12) $$c=\frac{r{\left(1+r\right)}^{n-1}}{{\left(1+r\right)}^{n}-1}\cdot \sum_{t=1}^{n}\left[P_{current}\cdot \frac{P_{t}^{E}}{P_{current}^{E}}\cdot q_{t}\right]$$

The calculation process of modes III and IV are the same as that of mode II.
